# EACVI survey on radiation exposure in interventional echocardiography

**DOI:** 10.1093/ehjci/jeae086

**Published:** 2024-04-18

**Authors:** E Galli, H Soliman-Aboumarie, L Gargani, P Szymański, A Gimelli, S E Petersen, L E Sade, I Stankovic, E Donal, B Cosyns, E Agricola, M R Dweck, N Ajmone Marsan, V Delgado, D Muraru

**Affiliations:** University of Rennes, CHU Rennes, Inserm, LTSI—UMR 1099, F-35000 Rennes, France; Department of Anesthetics and Critical Care, Harefield Hospital, Royal Brompton and Harefield Hospitals, Guy’s and St. Thomas NHS Foundation Trust, London, UK; School of Cardiovascular Sciences and Medicine, King’s College, London, UK; Department of Surgical, Medical and Molecular Pathology and Critical Care Medicine, University of Pisa—Pisa, Italy; Centre for Postgraduate Medical Education, Warsaw, Poland; Centre for Clinical Cardiology, National Institute of Medicine MSWiA, Warsaw, Poland; Department of Imaging, Fondazione Toscana Gabriele Monasterio, Via Moruzzi 1, 56124 Pisa, Italy; William Harvey Research Institute, Queen Mary University London, London, UK; University of Pittsburgh Medical Center, Heart and Vascular Institute, Pittsburgh, PA, USA; Faculty of Medicine, Clinical Hospital Centre Zemun, University of Belgrade, Belgrade, Serbia; University of Rennes, CHU Rennes, Inserm, LTSI—UMR 1099, F-35000 Rennes, France; Cardiology Department, Centrum voor Hart en Vaatziekten (CHVZ), Universitair ziekenhuis Brussel, Brussels, Belgium; Cardiovascular Imaging Unit, IRCCS San Raffaele Scientific Institute, Milan, Italy; BHF Centre for Cardiovascular Science, University of Edinburgh, Chancellors Building, Little France Crescent, Edinburgh EH16 4SB, UK; Department of Cardiology, Leiden University Medical Center, Albinusdreef 2, 2300 RC Leiden, The Netherlands; Department of Cardiovascular Imaging, Hospital Universitari Germans Trias i Pujol, Badalona, Barcelona, Spain; Department of Medicine and Surgery, University of Milano-Bicocca, Milan, Italy; Department of Cardiology, Istituto Auxologico Italiano, IRCCS, San Luca Hospital, Milan, Italy

**Keywords:** EACVI, survey, architectural shielding, interventional echocardiography, personal protection device, radiation exposure

## Abstract

**Aims:**

The European Association of Cardiovascular Imaging (EACVI) Scientific Initiatives Committee performed a global survey on radiation exposure in interventional echocardiography. The survey aimed to collect data on local practices for radioprotection in interventional echocardiography and to assess the awareness of echocardiography operators about radiation-related risks.

**Methods and results:**

A total of 258 interventional echocardiographers from 52 different countries (48% European) responded to the survey. One hundred twenty-two (47%) participants were women. Two-thirds (76%) of interventional echocardiographers worked in tertiary care/university hospitals. Interventional echocardiography was the main clinical activity for 34% of the survey participants. The median time spent in the cath-lab for the echocardiographic monitoring of structural heart procedures was 10 (5–20) hours/month. Despite this, only 28% of interventional echocardiographers received periodic training and certification in radioprotection and 72% of them did not know their annual radiation dose. The main adopted personal protection devices were lead aprons and thyroid collars (95% and 92% of use, respectively). Dedicated architectural protective shielding was not available for 33% of interventional echocardiographers. Nearly two-thirds of responders thought that the radiation exposure of interventional echocardiographers was higher than that of interventional cardiologists and 72% claimed for an improvement in the radioprotection measures.

**Conclusion:**

Radioprotection measures for interventional echocardiographers are widely variable across centres. Radioprotection devices are often underused by interventional echocardiographers, portending an increased radiation-related risk. International scientific societies working in the field should collaborate to endorse radioprotection training, promote reliable radiation dose assessment, and support the adoption of radioprotection shielding dedicated to interventional echocardiographers.

## Introduction

Over the last decade, the expanding number of complex structural heart procedures requiring advanced, real-time echocardiographic guidance has led to the development of interventional echocardiography as a specific subspecialty in the field of cardiac imaging.^1^

Interventional echocardiography is associated with important occupational exposure to X-rays. Among the three imaging techniques that involve radiation (X-ray fluoroscopy, X-ray CT, nuclear scintigraphy), X-ray fluoroscopy has the potential to deliver the largest dose of radiation to patients, operators, and nearby medical staff, depending on equipment quality and calibration, as well as the approach taken by interventional cardiologists. Compared to interventional radiologists and interventional cardiologists, interventional echocardiographers might be exposed to even higher doses of radiation, as they are often in the closest proximity to the patient and the X-ray source.^2–7^ The radiation dose received by interventional echocardiographers is generally highest to the exposed hand and lower body,^2^ as confirmed by computer simulations visualizing radiation exposure.^4^ This higher exposure is due to several factors which include the proximity of interventional echocardiographers to the radiation source and to the patient (who is by itself an important source of scattered radiation), the heterogeneity of the adopted radioprotection devices, the frequent lack of dedicated shielding, and the insufficient awareness of the radiation-related risk.^8^

Chronic occupational X-ray exposure has been associated with an increased risk of developing cataracts,^9^ leukemias, cancers,^10^ and also other diseases including early vascular and neurocognitive aging.^11^

Despite the universal recognition of the as low as reasonably achievable (ALARA) principle for radiation exposure and the basic safety standards laid down by the European Council Directive 2013/59/Euratom in 2018,^12^ the radiation risk of interventional echocardiographers often remains underappreciated.

Only a report by the American Society of Echocardiography published in 2014 provides generic indications to reduce radiation exposure in sonographers by remembering the four cardinal principles of radioprotection as decreasing the exposure time, increasing the distance from the radiation source, improving education in radioprotection and underscoring the importance of shielding. The same document also highlighted the importance of regular radiation safety training and pointed out the necessity of recognizing echocardiographers as a group of healthcare workers potentially exposed to medical radiation.^8^

The aims of the present Survey endorsed by the European Association of Cardiovascular Imaging (EACVI) Scientific Initiatives Committee were: (1) to collect data in Europe and worldwide on the radioprotection of interventional echocardiographers; (2) to evaluate the availability of dedicated personal protective devices and architectural shielding; (3) to assess the awareness of radiation-related risks; (4) to explore the impact of radiation exposure on pregnancy planning; and (5) to evaluate the perceived impact of motherhood on the career progression of interventional echocardiographers.

## Methods

The Survey was conducted by the EACVI Scientific Initiatives Committee from 10 October to 12 December 2024 and followed the established criteria for surveys of the international EACVI survey network.^13^

The survey was released on an online platform and consisted of 24 questions including single, multiple choice, and open questions addressed to all cardiac imagers performing interventional echocardiography to guide structural heart interventions across Europe and beyond. The invitation to participate in the Survey was disseminated via the EACVI newsletter and social media.

## Results

**Figure 1:**
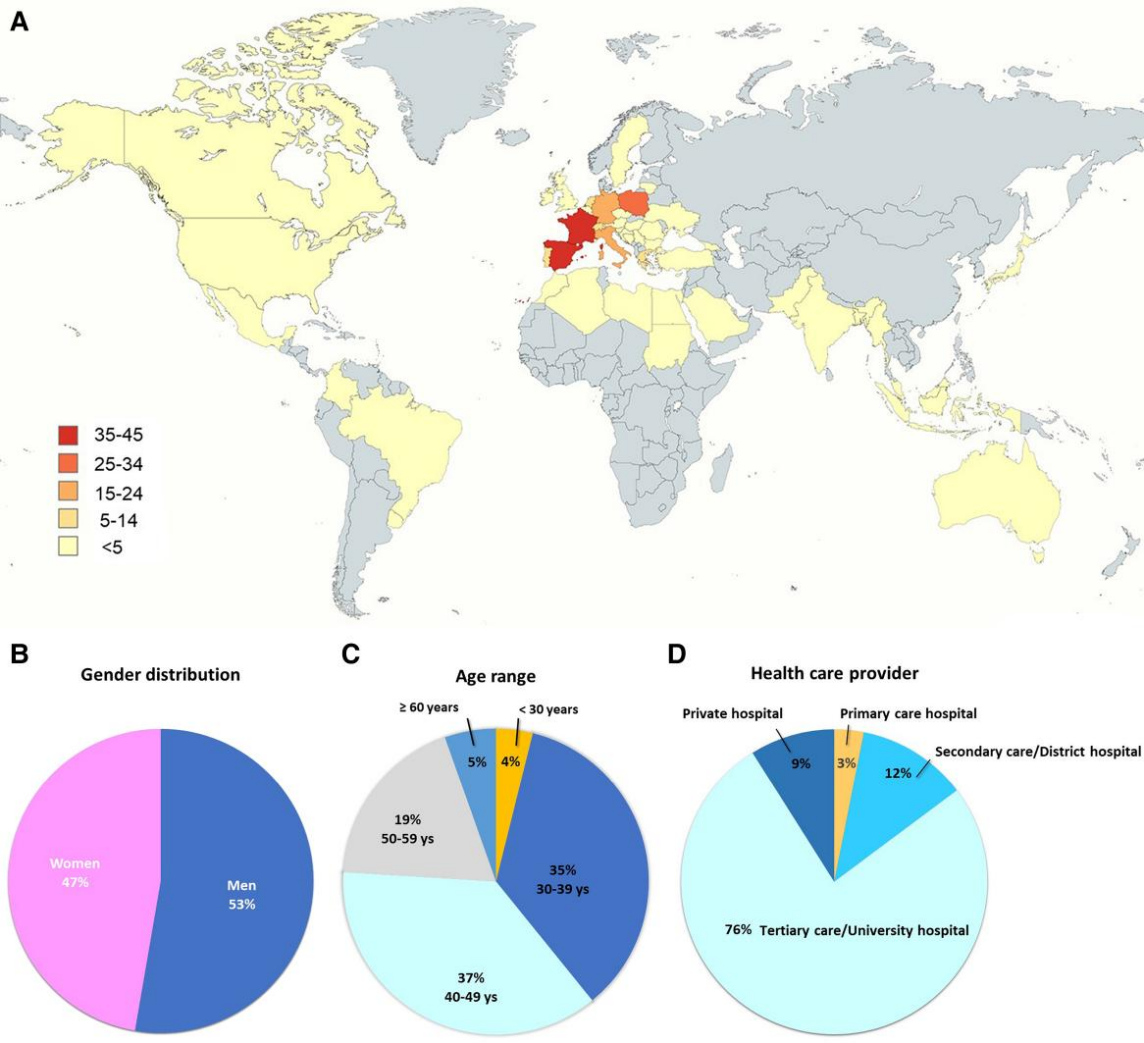
Distribution of survey participants by country (*A*), gender (*B*), age range (*C*), and work environment (*D*).

A total of 258 interventional echocardiographers from 52 countries participated in the survey. Two hundred eighteen participants (85%) were from Europe [Armenia (1), Austria (1), Belgium (4), Bulgaria (1), Croatia (3), Cyprus (1), Czechia (1), France (40), Germany (22), Greece (5), Ireland (1), Italy (24), Lithuania (1), Luxembourg (2), Monaco (1), Netherlands (5), Romania (2), Slovenia (3), Serbia (2), Spain (44), Sweden (2), Poland (28), Portugal (6), Switzerland (6), Ukraine (2), United Kingdom (4)], whereas 40 (15%) were from non-European countries [Bangladesh (2), Brazil (2), India (4), Canada (1), Egypt (1), Indonesia (2), Japan (4), Libya (1), Mexico (2), Morocco (1), Myanmar (1), Pakistan (2), Saudi Arabia (2), Singapore (1), Sudan (1), United States of America (2), and Uruguay (1)] (*Figure 1A*).

Gender distribution was quite evenly split, with only a slightly higher prevalence of men (53% vs. 47%, *Figure 1B*). The majority (72%) of interventional echocardiographers were 30 to 50 years old (*Figure 1C*). The survey’s participants came mainly from Tertiary Care/University Hospitals (76%), followed by secondary care (12%), private (9%) and primary care (3%) hospitals (*Figure 1D*).

### Interventional echocardiography activity

**Figure 2:**
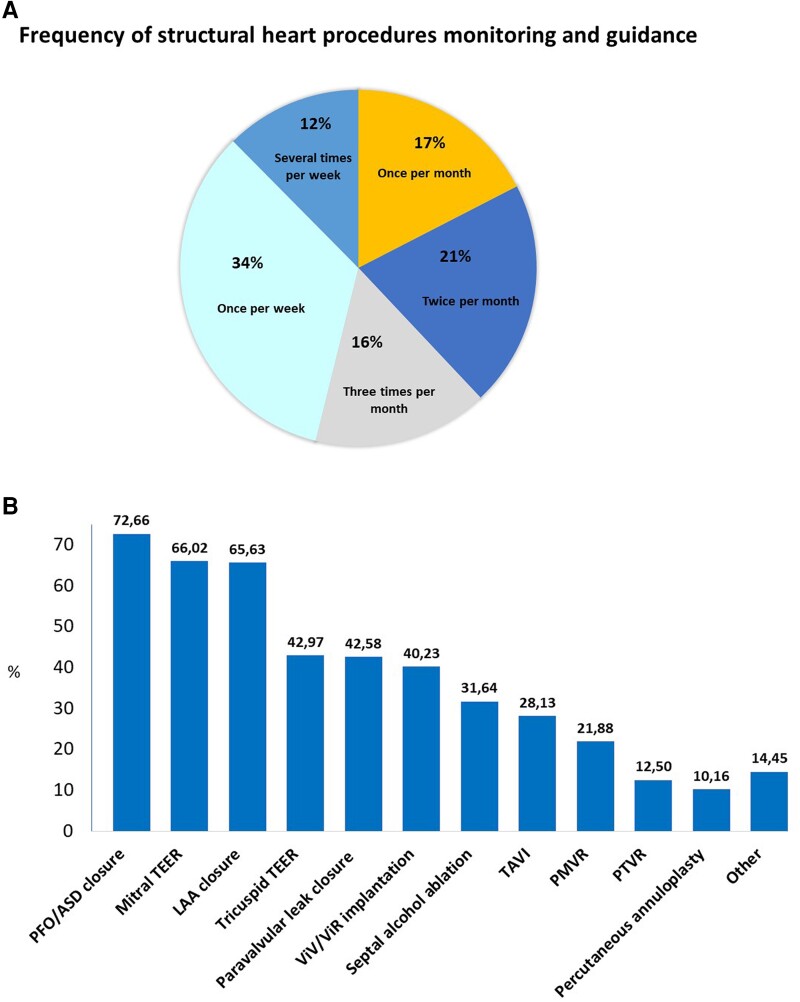
Interventional echocardiography activity. Time devoted to interventional echocardiography (*A*) and bar chart displaying the percentages of survey responders guiding the corresponding structural heart intervention (*B*). LAA, left atrial appendage closure; PFO, percutaneous foramen ovale; PMVR, percutaneous mitral valve replacement; PTVR, percutaneous tricuspid valve replacement; ViR, valve in ring; ViV, valve in valve.

sInterventional echocardiography was the main clinical activity for 34% of the participants, with 12% guiding structural heart procedures several times per week and 34% at least once per week (*Figure 2A*). The median time spent in the cath-lab each month was 10 (5–20) hours. The most frequently reported kind of procedures monitored by the participants were percutaneous closure of foramen ovale and atrial septal defects (ASDs) (73%), followed by left atrial appendage closure (LAA) (66%), and percutaneous mitral (66%) and tricuspid (43%) valve repair (*Figure 2B*).

### Monitoring of radiation doses, radiological protection training, and awareness of the occupational radiation risk

**Figure 3:**
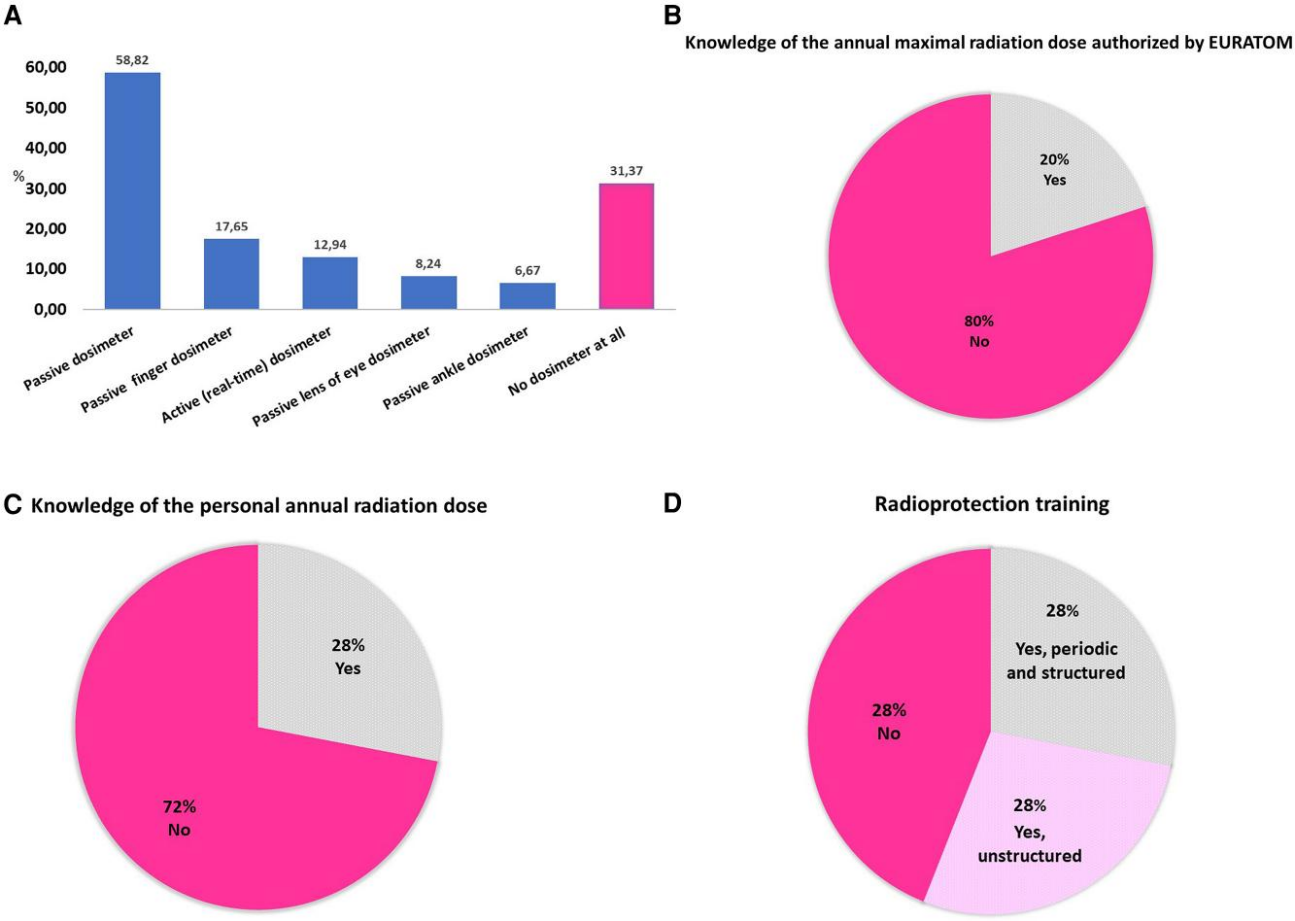
Monitoring of radiation doses. Type of dosimeters used by the survey’s responders (*A*), knowledge of the annual radiation dose limits authorized by EURATOM (*B*), knowledge of the personal annual radiation dose (*C*), and radioprotection training attendance (*D*).

To monitor radiation exposure, the survey’s responders relied essentially on passive chest dosimeters, which were adopted by 59% of interventional echocardiographers. Only 13% of the participants reported regular use of active (real-time or operational) dosimeters. Similarly, passive finger, lens eye and ankle dosimeters were worn only by 18%, 8%, and 7% of interventional echocardiographers, respectively. Importantly, 31% of the survey’s participants did not wear dosimeters during interventional procedures (*Figure 3A*).

Only 20% of interventional echocardiographers knew the maximal radiation doses authorized by the EURATOM^14^ and only 28% were aware of their estimated annual radiation exposure (*Figure 3B* and *C*).

Regular and structured radiological protection training was followed by only 28% of interventional echocardiographers. Twenty-eight percent of responders received some sort of non-structured education in radioprotection, whilst 43% did not have any training in radioprotection (*Figure 3D*).

Interestingly, the majority of interventional echocardiographers (65%) thought that the radiation exposure of the echocardiography operators was higher that than of interventional cardiologists, with a further 17% of that the exposure was similar to that of interventional cardiologists. Only 15% of participants considered the exposure of interventional echocardiographers lower than that of interventional cardiologists, whilst 3% had no specific opinion on this topic.

### Radioprotection devices availability

Concerning the use of wearable radioprotection devices, lead aprons and thyroid collars were used by nearly all interventional echocardiographers who answered the survey (95% and 92%, respectively).

**Figure 4:**
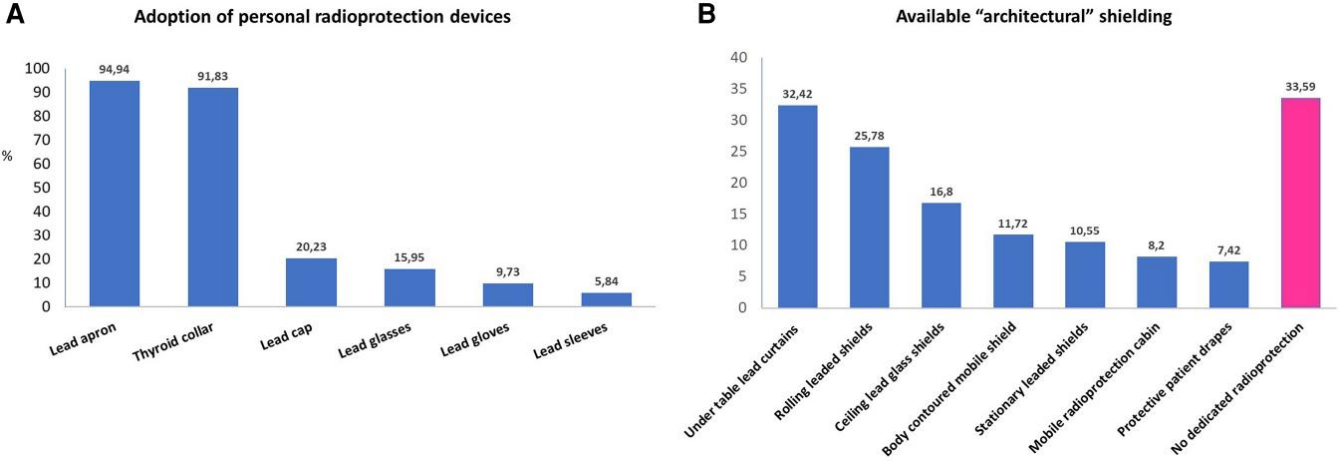
Personal (*A*) and architectural (*B*) radioprotection devices regularly adopted by the survey responders.

Lead caps, gloves, glasses, and sleeves were routinely used only in 20%, 16%, 10%, and 6% of cases, respectively (*Figure 4A*).

The availability of architectural or mounted shielding specifically applied for the radioprotection of the interventional echocardiographers was variable. Under-table curtains and rolling lead shields were available for 32% and 26% of the interventional echocardiographers, whereas body-contoured mobile radioprotective shields were available only in 12% of cases. Interestingly, 34% of the interventional echocardiographers did not have dedicated protective shielding during the guidance of structural heart procedure (*Figure 4B*).

Thirty-three percent of the survey’s participants were not able to look at the fluoroscopy screen during the procedures to see when the X-ray was active.

Finally, 72% of interventional echocardiographers considered that the radioprotective devices available in the cath-lab for echocardiography operators were not sufficient and needed improvement.

**Figure 5:**
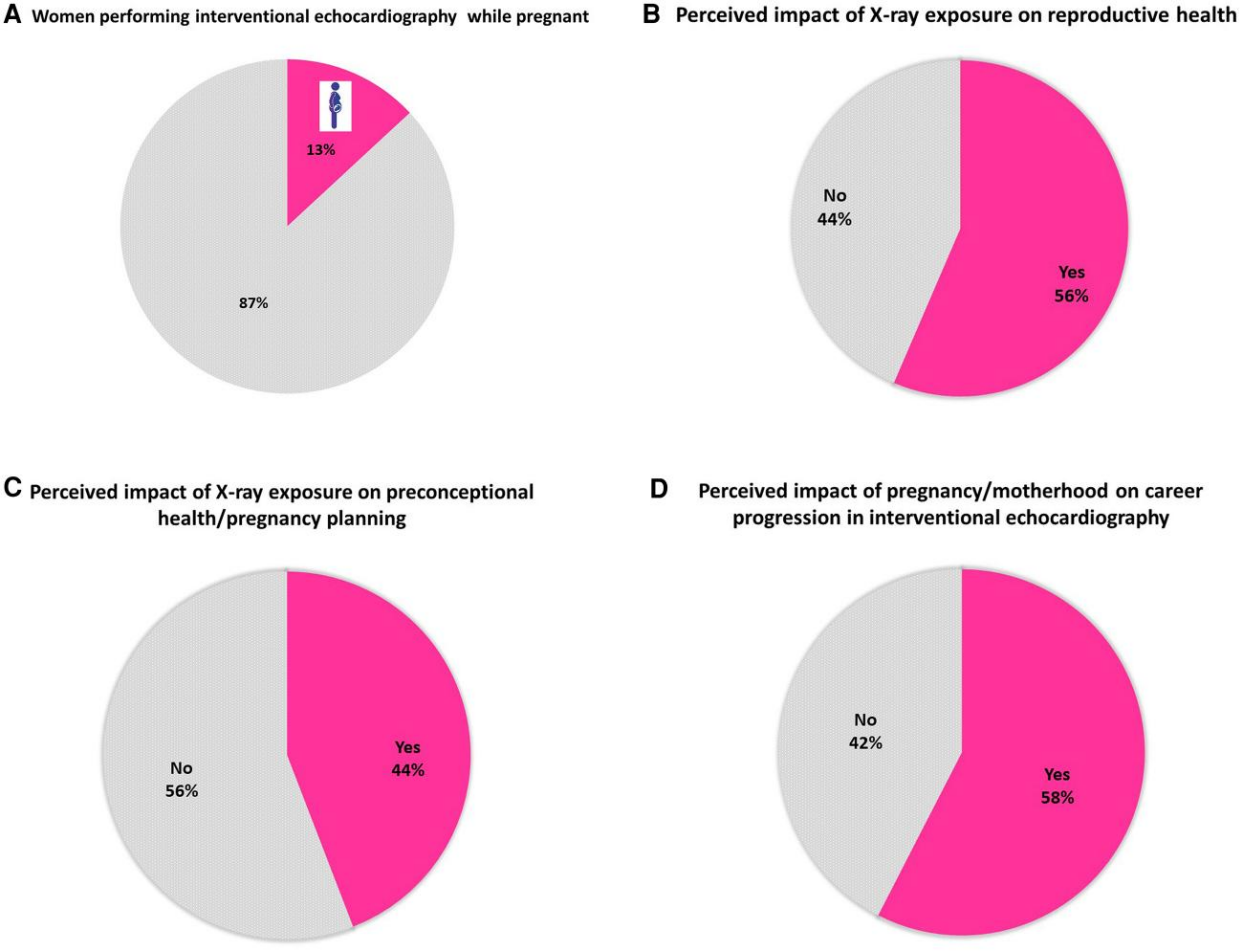
Impact of radiation exposure of reproductive health and pregnancy planning. Percentage of women participating in the survey and performing interventional echocardiography during pregnancy (*A*), perceived impact of radiation exposure on reproductive health (men and women involved) (*B*), perceived impact of an activity as interventional echocardiographer on periconceptional health/pregnancy planning (*C*), and perceived impact of pregnancy/motherhood on career progression in interventional echocardiography (*D*).

Whilst interventional echocardiography is generally avoided during pregnancy because of formal institutional regulations and/or personal decision making, 13% of women respondents described working as interventional echocardiographers during pregnancy (*Figure 5A*). The main special precautions applied by the host institution in these cases were wearing a double lead apron (1 answer), checking radiation exposure weekly (1 answer), and reducing the overall radiation dose (1 answer). Finally, 28% of the survey responders felt that special radioprotection precautions were necessary during pregnancy.

### Concerns regarding reproductive health, pregnancy, and career opportunities in interventional echocardiography

The impact of radiation exposure on reproductive health was a concern for 58% of interventional echocardiographers (men and women) answering the survey (*Figure 5B*).

Forty-four percent of women felt that their activity as interventional echocardiographers affected their preconception health and pregnancy planning (*Figure 5C*). On the other hand, 58% of women were concerned that maternity and subsequent parenthood might have negatively impacted their career as an interventional echocardiographer (*Figure 5D*).

## Discussion

This global survey provides a snapshot of the radiation exposure, radioprotection device availability, and radiation risk awareness of interventional echocardiographers.

### Demography and mainstream activity of interventional echocardiographers

As expected, interventional echocardiographers participating to the survey worked essentially in tertiary care/university hospitals. This relatively new subspecialty is essentially practiced by people who are in their early or mid-career phase (30–49 years), potentially having a long-lasting activity as interventional echocardiographers and subsequently a significant professional X-ray exposure. This aspect is even more relevant if we consider that interventional echocardiography was the main clinical activity for 34% of the survey’s responders, with one-third of interventional echocardiographers performing echocardiography to guide structural heart intervention at least once per week. The type of structural heart procedures guided by interventional echocardiographers was highly heterogeneous. This implies an intrinsic variability in radiation exposure, which is generally higher for new or challenging procedures, such as paravalvular leak closure and tricuspid repair/replacement, and decreases progressively for left atrial appendage closures, mitral transcatheter edge-to-edge repair (TEER), being the lowest for atrial septal defect and patent forame ovale closure.^2^

The convergence of more complex procedures towards high-volume referral centres might further accentuate the exposure of some interventional echocardiographers. Adequate radioprotection for interventional echocardiographers becomes therefore a major challenge for the safety and future development of this specific profession.

### Awareness of the radiation-related risk

In this survey, we considered four specific elements to assess the appreciation of the radiation-related risks for interventional echocardiographers: (i) the extensive and appropriate use of passive and active dosimeters; (ii) the systematic participation in radioprotection training; (iii) the knowledge of the regulatory limits established by the European Atomic Energy Community (EURATOM) for radiation occupational exposure^14^; (iv) the knowledge of an operator’s annual radiation exposure.

The monitoring of radiation exposure can be obtained by either passive or active dosimeters. As recommended by the International Commission on Radiological Protection (ICRP),^15^ passive dosimeters should be used to record the annual and effective radiation dose at different sites (e.g. eye lens, chest, ankle, finger). On the other hand, active or real-time dosimeters are useful to assess individual exposure on a day-by-day basis.^15,16^

Our survey shows that half of the interventional echocardiographers wear passive chest dosimeters during their activity in the cath-lab, whereas the use of eye and extremity passive dosimeters is much lower (17% for the eye of lens dosimeter to 7% for ankle dosimeter). This can cause a significant underestimation of the effective radiation dose for interventional echocardiographers, as previous reports demonstrated higher radiation doses for the head, arm, and lower body for echo operators guiding structural heart procedure.^2,4^

Although the use of active dosimeters is recommended to monitor radiation exposure during new or specific procedures to optimize radiation exposure and/or for educational purposes, this kind of dosimeter is largely underused by interventional echocardiographers in current clinical practice. Finally, one-third of interventional echocardiographers who participated in the survey did not wear a dosimeter during their activity in the cath-lab.

This observation reflects negligence in the application of the basic radioprotection measures endorsed by International societies^12,14,15,17,18^ and underscores a potentially dangerous underappreciation of the radiation-related risk for interventional echocardiographers. This lack of awareness of the radiation risk-related to the monitoring of structural heart procedures is also mirrored by the fact that nearly 30% of interventional echocardiographers think that their exposure is similar to or lower than that of interventional cardiologists.

Despite the importance of a safety culture for professionals performing interventional procedures^12,14,15^ and the relevance given to radiological protection from Institutions working in the field,^17,18^ only one-third of interventional echocardiographers answering this survey received a structured and periodic training certification in radioprotection by their hosting institution. The remaining survey participants did not receive structured teaching or had no education at all.

Given that echocardiography does not involve ionizing radiation, the formal training of echocardiographers (including interventional echocardiographers) primarily focuses on the principles of ultrasound, and, as a result, their familiarity with the specifics of radiation safety might be less comprehensive compared to radiologists or cardiologists who regularly perform or interpret X-ray-based imaging. Hospital safety protocols and training requirements related to radiation safety are specifically targeted at personnel who are responsible for the administration of ionizing radiation; thus, in some institutions, the interventional echocardiographers, despite being exposed to X-rays, may not be formally required to undergo this training before embarking on this activity.

This lack of radioprotection education is reflected in the low percentage (20%) of interventional echocardiographers knowing the maximal annual radiation dose authorized by the EURATOM,^14^ with only one-third of interventional echocardiographers being aware of their own personal estimated annual radiation exposure.

### Personal or architectural protection device use and availability

Personal protection by shielding devices is the pillar to limit X-ray exposure during procedures in the cath-lab according to the ALARA principle.

Our survey revealed that lead aprons and thyroid collars were largely used by interventional echocardiographers. However, the head, hands, arms, and legs, which are highly exposed to X-ray during interventional echocardiography, cannot be adequately protected by the apron. Despite specific personal protection devices such as lead glasses, caps, sleeves, and gloves being effective in reducing irradiation, they were only worn by 6–20% of interventional echocardiographers participating in this survey.

Moreover, because of the position of interventional echocardiographers during structural heart procedures, dedicated architectural shielding is often necessary to significantly reduce the irradiation of echo operators.

Previous studies have shown that ceiling-suspended lead shields^5,6^ and lead curtains^6^ positioned on the side of the interventional echocardiographers can significantly reduce irradiation. The use of mobile lead cabins can further reduce interventional echocardiographers’ exposure, providing full-body protection for the interventional echocardiographer from X-ray exposure.^19^

However, many cath labs and hybrid rooms are still designed and organized to suit the needs of interventional cardiologists rather than other members of the structural heart team. Indeed in our survey, up to one-third of interventional echocardiographers declared not to have any dedicated protective architectural shielding. Consequently, 72% of survey responders felt that the available radioprotection devices were often insufficient and that there was a need for improvement in radioprotection measures for interventional echocardiographers.

### Pregnancy and reproductive concerns related to interventional echocardiographers’ activity

The real-life radiation exposure of interventional echocardiographers can vary according to the procedure and the organization of the operatory theatre. However, the radiation scattering of the bottom edge of the patient bed is associated with a higher exposure of the pelvic area compared to the thorax, as shown by either direct measurements or computer simulations.^4,20^

In our survey, nearly half of the participants were worried about the potential impact of radiation exposure on their reproductive health.

This might be particularly important for women performing interventional echocardiography in their childbearing age, because of the significant irradiation of the ovaries during the procedures.

Despite these concerns, the present survey did not show significant gender differences in access to interventional echocardiography, with a similar percentage of men and women performing this activity.

However, nearly one-third of women felt that the practice of interventional echocardiography affected their preconception health and their pregnancy planning and more than half perceived that their pregnancy/motherhood negatively impacted their career as interventional echocardiographer.

These aspects might, therefore, contribute to the development of a ‘career gap’ in the field of interventional echocardiography, as already observed for women in the field of interventional cardiology.^21^

Moreover, in the case of pregnancy, the practice of interventional echocardiography might be problematic because of the risk of foetal irradiation.

In our survey, only 13% of women continued their activities as interventional echocardiographers during pregnancy. In case of pregnancy, specific precautions to limit irradiation were taken by the hosting institution in 11% of cases. According to the open answers we received, stopping all the activities associated with X-ray exposure was the main measure taken, followed by wearing a double lead apron, weekly dose checks and reducing fluoroscopy exposure.

Several recent consensus documents in the field of interventional cardiology,^22^ interventional radiology,^23^ and electrophysiology,^24^ as well as recommendations from EURATOM^14^ and the International Commission on Radiological Protection^17^ underscore that an overall foetal radiation dose <1 mSv during pregnancy is acceptable, with a risk of malformation or cancer of 4.07%, similar to that of the general population.^24^ Nevertheless, until now, no specific data are available concerning the radiation exposure of pregnant women performing interventional echocardiography. As a consequence, women working as interventional echocardiographers in their childbearing age should be aware that their radiation exposure is probably higher than those of interventional cardiologists, and that radiation protection measures are less well-established for their working category, compared to interventional cardiologists.

Because of the great sensitivity of this issue, it seems reasonable that the decision on whether to continue activity as an interventional echocardiographer during pregnancy is left to the pregnant medical doctor, as recommended by the ICRP.^17^

According to European directives, when pregnant workers are exposed to radiation, employers also have a responsibility to assess the related risks and apply all the necessary protective measures,^14^ whilst avoiding professional discrimination in women in the childbearing age.^18^

As the figure of interventional echocardiographer is becoming more common and established for clinical practice, cardiologists aiming to select echocardiography as their main subspecialty should be educated about radiation hazards involved with intraprocedural guidance so that they make an informed decision about whether this is an aspect of their career they wish to pursue.

### Limitations

The survey reflects the answers of a limited number of voluntary participants and may suffer from sampling bias. Also, the interventional echocardiographers’ activities were highly heterogeneous in terms of duration and complexity, which might also affect the interpretation of our results. The different perceptions or interpretations of the questions by the responders can also influence the results of the present survey.

## Conclusions

Our data demonstrate wide variability and often underuse of radioprotection devices, highlighting the need for dedicated larger studies and specific actions, guided by both Scientific Societies and International Radioprotection Committees. Structured and regular training appears fundamental to educate interventional echocardiographers about radiation hazards, to instill best practices according to the ALARA principles and to ensure their commitment to maximizing protection. The assessment of the radiation dose should be extensively performed by either passive or active dosimeters. Data of previous studies also suggest that ankle, hand, and eye lens dosimeters might be fundamental to a because of the higher exposure of the extremities and the eyes.

Interventional and hybrid rooms should be specifically designed to offer dedicated protection to interventional echocardiographers by the application of appropriate shielding. Preventive measures should be applied to avoid discrimination and gender gap for interventional echocardiographers. Interdisciplinary education and collaborative efforts within the medical community should enhance awareness and ensure the safety of interventional echocardiographers.

## Data Availability

The data underlying this article are provided by the European Association of Cardiovascular Imaging (EACVI) by permission. Data will be shared on request to the corresponding author with permission of the EACVI.
